# West Nile Virus Prevalence across Landscapes Is Mediated by Local Effects of Agriculture on Vector and Host Communities

**DOI:** 10.1371/journal.pone.0055006

**Published:** 2013-01-30

**Authors:** David W. Crowder, Elizabeth A. Dykstra, Jo Marie Brauner, Anne Duffy, Caitlin Reed, Emily Martin, Wade Peterson, Yves Carrière, Pierre Dutilleul, Jeb P. Owen

**Affiliations:** 1 Department of Entomology, Washington State University, Pullman, Washington, United States of America; 2 Washington State Department of Health, Olympia, Washington, United States of America; 3 Department of Entomology, University of Arizona, Tucson, Arizona, United States of America; 4 Department of Plant Science, McGill University, Macdonald Campus, Ste-Anne-de-Bellevue, Quebec, Canada; University of Texas Medical Branch, United States of America

## Abstract

Arthropod-borne viruses (arboviruses) threaten the health of humans, livestock, and wildlife. West Nile virus (WNV), the world’s most widespread arbovirus, invaded the United States in 1999 and rapidly spread across the county. Although the ecology of vectors and hosts are key determinants of WNV prevalence across landscapes, the factors shaping local vector and host populations remain unclear. Here, we used spatially-explicit models to evaluate how three land-use types (orchards, vegetable/forage crops, natural) and two climatic variables (temperature, precipitation) influence the prevalence of WNV infections and vector/host distributions at landscape and local spatial scales. Across landscapes, we show that orchard habitats were associated with greater prevalence of WNV infections in reservoirs (birds) and incidental hosts (horses), while increased precipitation was associated with fewer infections. At local scales, orchard habitats increased the prevalence of WNV infections in vectors (mosquitoes) and the abundance of mosquitoes and two key reservoir species, the American robin and the house sparrow. Thus, orchard habitats benefitted WNV vectors and reservoir hosts locally, creating focal points for the transmission of WNV at landscape scales in the presence of suitable climatic conditions.

## Introduction

Viruses transmitted by arthropods (arboviruses) threaten the health of humans, livestock, and wildlife worldwide [1], [2]. Most arboviruses cycle primarily between blood-feeding arthropod vectors and wild vertebrates, and can subsequently be spread to incidental hosts such as humans or livestock [1], [2]. Diseases associated with arboviruses include dengue fever, yellow fever, West Nile encephalitis, and Chikungunya disease, all of which can cause severe symptoms and/or fatality in humans and other hosts.

Arbovirus transmission is governed by the ecological interactions between vectors, hosts, and pathogens across landscapes [1]. Focal points of infection develop in areas where populations of competent vectors, reservoir hosts, and susceptible recipient hosts interact. These focal points are often ephemeral, leading to dramatic fluctuations in the prevalence of some diseases over space and time [1], [2]. Understanding the complex set of factors that lead to the formation of focal points of infection, and subsequent disease spread across landscapes, is therefore essential for predicting and mitigating disease outbreaks [1].

As the causal agent of West Nile encephalitis, West Nile virus (WNV) is the most widespread arbovirus in the world [3]. In the United States, WNV was detected in New York State in 1999 and rapidly spread across the country. The invasion of the United States by WNV has caused regional declines of multiple bird species [4] and thousands of infections and deaths in humans and horses [3]. At a landscape scale, where infections per county have been analyzed, urbanization and agricultural intensification appear to increase the prevalence of WNV infection in humans and horses [5]–[7]. Increased temperatures and decreased precipitation have also been linked to increased infections [6], [8], [9]. However, although the prevalence of West Nile virus is assumed to be strongly affected by vector and host distributions [10], it remains unclear how local (i.e., sub-county level) interactions between WNV vectors and hosts are affected by land-use and climate to create focal points for WNV transmission across landscapes.

The work reported here had two objectives. First, we used spatially-explicit models to test whether land-use and climate affected the prevalence of WNV infection at landscape and local spatial scales. Second, to examine the mechanisms by which land-use and climate affect focal points of infection for WNV spread, we assessed the distributions of mosquitoes and birds involved in the transmission cycle. Thus, we first determined if factors associated with WNV infection were different, or not, depending on spatial scale. By subsequently analyzing communities of WNV vectors and reservoirs, we linked local vector and host distributions with the prevalence of WNV infection across landscapes.

## Methods

### Prevalence of West Nile Virus Infection

We examined the effects of land-use and climate on the prevalence of WNV infection at two main spatial scales: 1) landscape: the prevalence of WNV infections per county in humans, horses, and birds over Idaho (ID), Oregon (OR), and Washington (WA) states during 2007–2010 and 2) local: the prevalence of WNV in mosquitoes at field locations over eight counties in WA during 2009–2010. No specific permits were required for the described field studies. Data at the landscape scale were collected from the Center for Disease Control ArboNet database and the WA Department of Health database [11], [12].

At the local scale, data on the prevalence of WNV in mosquitoes (*Culex pipiens* and *Cx. tarsalis*) were collected at 101 and 108 field locations in 2009 and 2010, respectively. The locations of these sites were determined by respective mosquito control districts based on public input (complaints about mosquitoes) or their assessment of risk. All field sites were located on public land, and no specific permissions were required for these sampling activities. At each site, mosquitoes were collected using Encephalitis virus surveillance (EVS) traps baited with dry ice. The location of each trap was recorded with a Global Positioning System unit. Mosquitoes were trapped from 21 April to 7 October in 2009, and from 15 April to 22 September in 2010. The number of traps collected varied among locations (range 1–90). Variation in trap density was not associated with the prevalence of WNV at any particular location, but instead was based on methodology of the respective mosquito control districts and the accessibility of the field locations. For data analysis, we only included the locations with at least 5 traps, a condition met at 54 and 69 field locations in 2009 and 2010, respectively.

Collected bags of mosquitoes from EVS traps were kept in coolers until they were processed. For processing, mosquitoes were knocked down with dry ice and then sorted on ice or a chill table. The total number of female mosquitoes collected was recorded, and all female mosquitoes were pooled according to species (12–50 specimens per pool) prior to testing for WNV. We only included females in mosquito pools as only females blood-feed and are responsible for WNV transmission. Female mosquitoes were identified to species using a clear dichotomous key [13] by trained technicians at respective mosquito control districts. Identification of mosquitoes to species was necessary so that tested pools only contained *Cx. tarsalis* and *Cx. pipiens*, as these species account for the majority of WNV infections in the Pacific Northwestern United States [3]. Other mosquito species are not important vectors of WNV in our sampled region, and were therefore excluded from analyses. These data were also used in the analysis of mosquito abundance (see Vector and host distributions). When traps had more than 50 mosquitoes, a random subsample of 50 was used for WNV testing.

Mosquito pools were examined for the presence/absence of WNV RNA with the Rapid Analyte Measurement Platform (RAMP®) WNV test (Response Biomedical Corp., Burnaby, Canada), following the manufacturer’s instructions or by reverse transcription-polymerase chain reaction (RT-PCR). RAMP test results with a value of ≥300.0 RAMP units were considered positive. Test results with values between 50.1 and 299.9 RAMP units were considered negative, unless confirmed positive by PCR testing (these samples were shipped to Oregon State University for confirmatory testing). Samples with values <50 RAMP units were considered negative and were not tested by PCR. Mosquito samples tested by PCR only were shipped to the Center for Vector-borne Diseases at the University of California, Davis. The detection of WNV RNA was conducted with real-time -PCR, using TaqMan Fast Virus 1-Step Master Mix (Applied Biosystems, Carlsbad, USA) with WNV specific primers [14] on an ABI MagMax instrument.

### Vector and Host Distributions

To link the prevalence of WNV infections with vector and host distributions, we examined local factors affecting mosquito and bird communities. Data on mosquito abundance were taken from the WNV survey sites. Data on bird abundance and species composition were obtained from 136 Breeding Bird Survey (BBS) sites from 2007 to 2010 [15] (Fig. S1). The BBS is a United States Geographical Survey funded project that examines bird communities throughout the United States. The BBS follows a standard protocol, with observers driving along a 39.4-km roadside route. Every 0.8 km, observers record the total number and species of birds seen or heard during a 3-min observation period. The total area sampled per route is 25.4 km^2^. Survey routes are sampled once per year. At each BBS site, we calculated the total bird abundance, the number of bird species, and the abundance of two common enzootic amplification hosts of WNV: the American robin and the house sparrow [16]–[18].

### Land Cover and Climatic Data

We obtained land cover and climatic data to relate land-use and climate with the prevalence of WNV infections and the distributions of vectors and hosts. We determined land cover using USDA Cropland Datalayer (CDL) maps, which provide remotely sensed data on land-use throughout the United States [19]. From 2007–2009, CDL maps were produced at a 56-m resolution; 2010 maps were produced at a 30-m resolution. These differing resolutions, however, did not affect how land cover was evaluated.

To determine land cover for the landscape analysis, we imported the CDL maps into ArcGIS [20] and then calculated the abundance of three habitat types in each county over WA, OR, and ID: vegetable/forage crops, orchards, and natural (Table S1). Vegetable/forage crops were considered differently from orchards because they are typically grown under central-pivot irrigation, while orchards are not. To determine land cover surrounding each BBS site, we followed the methods of Meehan et al. [21]. Briefly, we extracted the area for each habitat type from rectangular buffers around survey routes, with buffers extending 0.4 km from the route to reflect the observation distance at which bird species were surveyed. To scale habitat area derived from the rectangular buffer (31.5 km^2^) to the sum of circular buffers sampled by the BBS (25.4 km^2^, see above), we multiplied land cover areas by the factor 0.81 (25.4 km^2^/31.5 km^2^). This scaling assumes that land-use contained within the rectangular buffers is the same as in the area sampled by the BBS observer [21].

To determine land cover surrounding mosquito trapping locations, we drew 10 concentric rings in GIS around each field location over a scale from 0.05 to 0.5 km (each ring was 0.05 km). The maximum radii was based on usual mosquito dispersal, which typically occurs over distances <0.5 km [22]. Furthermore, the use of distances >0.5 km did not improve the fit of models during data analysis, indicating that variation in habitat structure did affect mosquito over these distances. In each ring, we measured the acreage of each of the three habitat types (vegetable/forage, orchard, natural) using ArcGIS.

Climatic data (temperature and precipitation) were collected from the Western Regional Climate Center [23]. To obtain climatic data for the landscape analysis, we randomly selected three weather stations from each county where WNV had been detected. From each station, we obtained the annual average temperature and precipitation across the sampling period (2007 to 2010) to determine the average climatic conditions over the period where WNV was surveyed. The obtained climatic data were averaged over these stations to produce county averages; in cases where only one or two stations were located in a county, data from those stations were used.

To assess climatic factors associated with our mosquito and bird sites, we used data from these same weather stations. In these analyses, we estimated the temperature and precipitation at field locations where mosquitoes where trapped or at BBS sites (at the center of BBS survey rectangles), using inverse distance weighting (IDW) interpolation in ArcGIS [20]. Here, IDW was used to interpolate climatic data between spatially discontinuous weather stations. For each field location, the temperature and precipitation for each year (2007–2010) was calculated by averaging the weighted sums of temperature and precipitation data from the 12 nearest weather stations, the stations farther away influencing the climate estimates less than those closer to the site (decay component = 2).

### Data Analyses

At the landscape scale we used multiple regression models to evaluate associations between the three habitat type acreages (vegetable/forage crops, orchards, and natural), two climatic variables (temperature and precipitation), and all two-way interactions, on the prevalence of WNV infections in humans, horses, and birds. Prior to data analysis, the number of WNV infections in humans was standardized by county population; infections in horses and birds were standardized by county area. WNV prevalence in each of the three groups (human, horse, bird) was highly non-normal, so we used rank-based statistics which do not require the normality assumption [24]. Each county served as one sampling unit in analyses performed at the landscape scale. For each model, we first used stepwise regression [24] to select a subset of explanatory variables that minimized the Akaike’s Information Criterion (AIC); these models were subsequently used in all further analyses of factors affecting the prevalence of WNV infection. Results obtained with the Bayesian Information Criterion (BIC) were very similar and therefore are not reported. These models were fit in JMP [25].

We used logistic regression to evaluate relationships between habitat type acreages, climatic variables, and the local prevalence of WNV infection in mosquito pools. WNV infection counts were binomial, with each field location where mosquitoes were trapped providing one observation. The number of mosquitoes tested per pool was included as a covariate. Models were fitted separately in 2009 and 2010, at each of the 10 spatial scales (0.05–0.5 km). In each year, we used the AIC and the corresponding *R* ^2^ value to determine the scale at which the most variation was explained, and used these models for further analyses. To assess the presence of spatial autocorrelation in the residuals from the logistic regression models selected in each year analyzing the prevalence of WNV in mosquitoes, we used the co-regionalization analysis with a drift method [26]–[28], and Pearson’s chi-square residuals and deviance residuals were evaluated before and after applying a Box-Cox transformation­. In this context (i.e., geostatistical analysis of spatial data at small vs. large scale), it is possible to model a ‘drift’ (representing large-scale heterogeneity of the mean) globally or using a moving window. These models were fit in Matlab [29]. From the 16 spatial autocorrelation analyses (2 years × 2 types of residuals × 2 data transformations × 2 drift models), only one (2010, Pearson residuals, no Box-Cox transformation, local drift model) revealed significant spatial autocorrelation at relatively small distances (up to 6 km). There was thus no need for adjusting the statistical tests of significance of the logistic regression models and estimated slopes. Resulting logistic regression models were fit in JMP [25].

For analysis of mosquito and bird abundance, and bird species richness, we used multiple regression models. Each field location where mosquitoes were trapped or each BBS site provided one observation. In these models, we used rank-based statistics that did not require the normality assumption [24]. For mosquito abundance, models were analyzed at each of 10 spatial scales (from 0.05 to 0.5 km), with land-use at each scale as an explanatory variable (climate was the same at all scales). For birds, abundance and species richness at each BBS site were averaged over sampling years, and a single value of land-use from GIS models was used in the analyses. Semivariograms were computed to quantify and analyze spatial autocorrelation [30] in the abundance of mosquitoes and the abundances and species richness of bird hosts. Spatial autocorrelation was accounted for in tests of significance following the approach of Carrière et al. [30], by using effective sample sizes and degrees of freedom in modified *t* tests designed for multiple regression analyses with spatial data [28]. These analyses were performed in Matlab [29].

## Results

### Prevalence of West Nile Virus Infection

**Figure 1:**
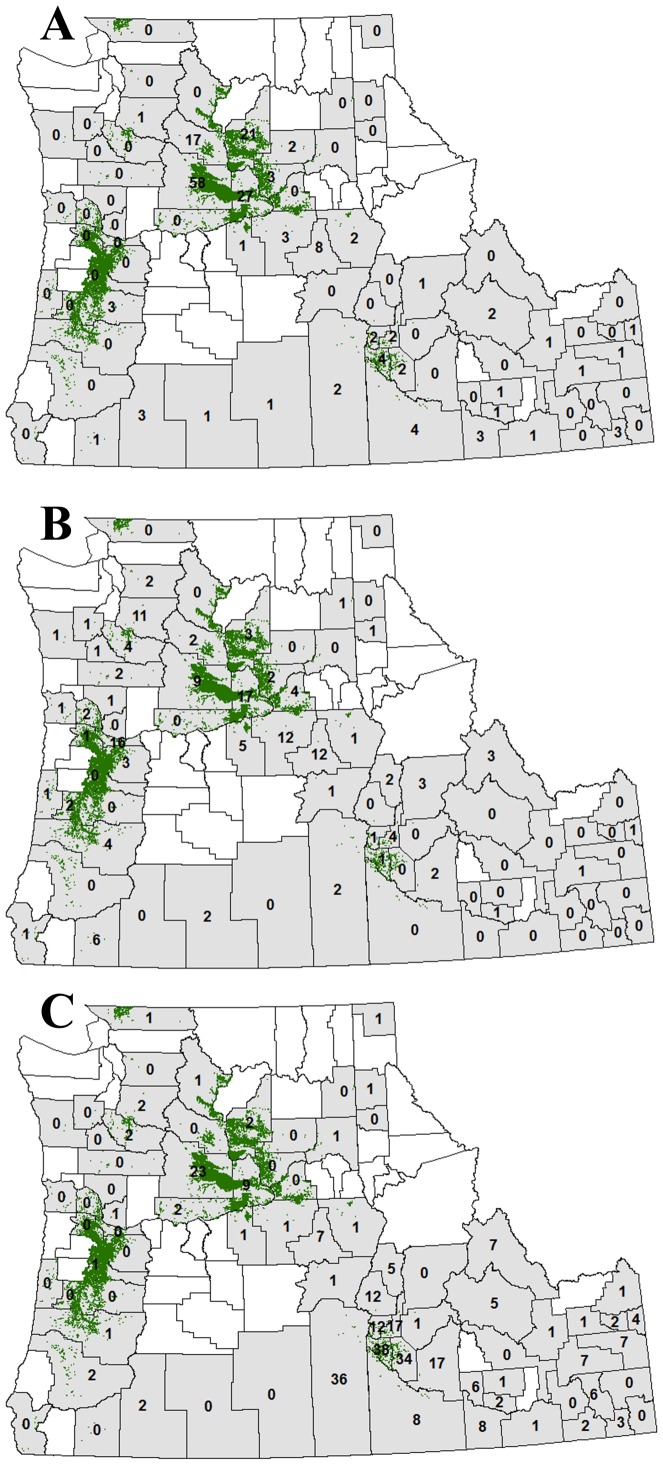
Land-use and the prevalence of West Nile virus infection. Number of West Nile virus (WNV) infections in (A) horses, (B) birds, and (C) humans from 2007 to 2010 over Idaho, Oregon, and Washington States. Counties shaded gray had at least one WNV case detected in any species over this period, with the numbers indicating the number of infections in each organism(s). Land covered by orchard habitats is shown in green.

**Figure 2:**
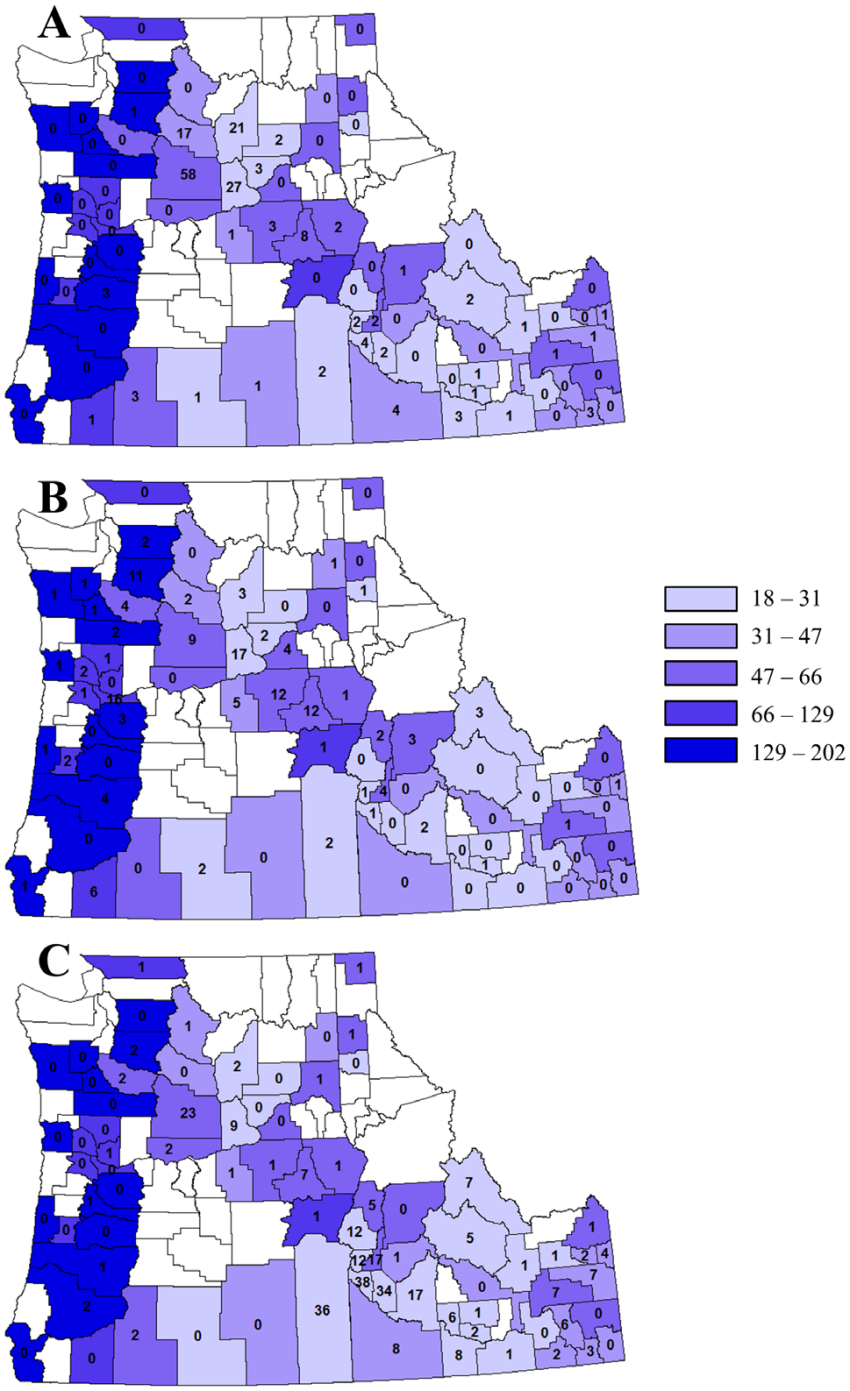
Precipitation and the prevalence of West Nile virus infection. Number of West Nile virus (WNV) infections in (A) horses, (B) birds, and (C) humans from 2007 to 2010 over Idaho, Oregon, and Washington States. Counties are shaded according to their annual average precipitation (mm, only counties where WNV was detected in any species are shaded), with the numbers indicating the number of infections in each organism(s).

At the landscape scale, the numbers of WNV infections in horses and birds were significantly positively associated with the acreage of orchard habitats, but were not significantly affected by other habitats (Fig. 1a,b, Table S2). The number of human infections was not significantly associated with any habitat (Fig. 1c, Table S2). The number of infections in humans, horses, and birds was significantly negatively associated with precipitation (Fig. 2, Table S2), and the interaction between orchard acreage and precipitation was significantly negative (Table S2). Temperature was not significantly associated with the number of infections in humans, horses, or birds (Table S2).

**Figure 3:**
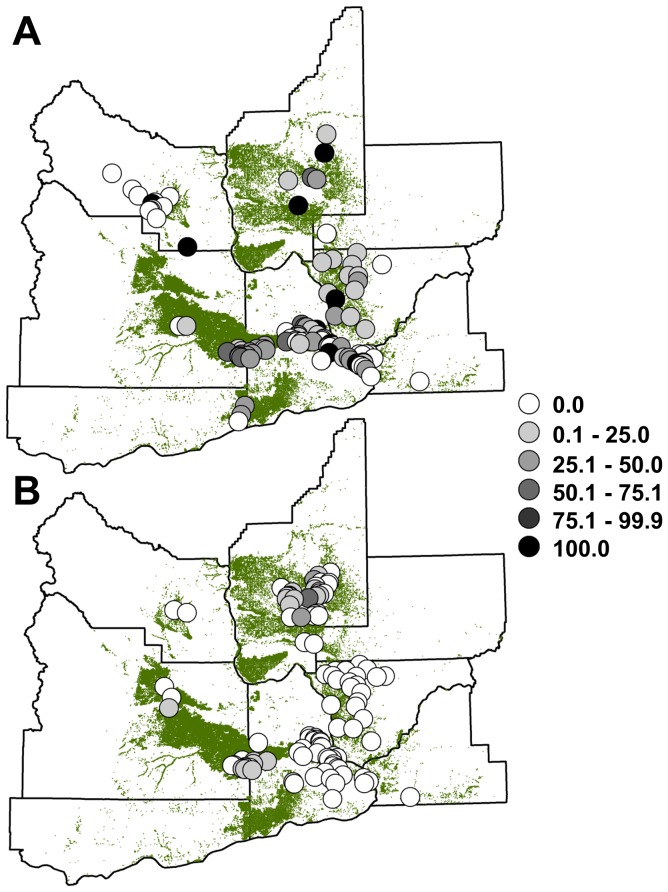
Prevalence of West Nile virus infection in mosquitoes. Proportion of mosquito pools that tested positive for West Nile virus (WNV) infection at each mosquito trap location in (A) 2009 and (B) 2010 across eight counties in Washington State. Each mosquito trapping location is indicated with a circle, with different levels of shading indicating a different class of values for the proportion of positive mosquito pools. Land covered by orchards in each year is shown in green.

At a local scale, in both 2009 and 2010 the prevalence of WNV infections in mosquitoes was significantly positively associated with the acreage of orchard habitats (Fig. 3, Table S3). These results were based on a total of 22,141 and 28,504 *Cx. pipiens* collected and tested in 2009 and 2010, respectively; a total of 25,461 and 49,293 *Cx. tarsalis* were collected and tested in both years, respectively. The prevalence of WNV infection was similar in both species, with 14.5 or 13.5% of mosquito pools containing *Cx. pipiens* or *Cx. tarsalis* testing positive for WNV, respectively. In 2010, the prevalence of WNV in mosquitoes was also positively associated with the acreage of vegetable/forage and natural habitats, and negatively associated with temperature (Table S3). The prevalence of WNV in mosquitoes was not significantly associated with precipitation in either year (Table S3). In both years, the strength of these effects varied from 0.1–0.5 km (Figs. S2, S3).

#### Effects of land-use and climate on mosquito and bird distributions

The abundance of both *Cx. pipiens* and *Cx. tarsalis*, and the combined abundance of these two species, increased locally with greater acreage planted to orchards, but were unaffected by other habitat types or climatic variables (Table 1). Similarly, the abundance of American robins and house sparrows increased at sites with greater orchard acreages (Table 1). While American robins and house sparrows benefited from orchard habitats, we found no evidence that land-use or climate affected total bird abundance or species richness (Table 1). Thus, American robins and house sparrows increased in relative as well as absolute abundance in orchards (Table 1).

**Table 1:** Results of multiple regression analysis.

Response	Explanatory Variable									
	Temperature		Precipitation		Vegetable/forage		Orchard		Natural	
	Slope	*P*	Slope	*P*	Slope	*P*	Slope	*P*	Slope	*P*
*Cx. pipiens* abundance	−0.40	0.34	−0.24	0.51	−0.16	0.91	0.58	0.0002	0.082	0.51
*Cx. tarsalis* abundance	0.017	0.95	0.16	0.58	0.27	0.17	0.34	0.034	0.23	0.26
*Cx pipiens*+*Cx tarsalis* abundance	0.028	0.91	0.24	0.30	0.17	0.12	0.51	<0.0001	0.088	0.39
American robin abundance	0.039	0.45	0.10	0.41	−0.17	0.26	0.22	0.053	−0.063	0.69
House sparrow abundance	0.011	0.89	−1.02	0.38	−0.11	0.42	0.30	0.0036	−0.12	0.40
Robin+sparrow abundance	0.062	0.50	0.034	0.79	−0.15	0.33	0.22	0.046	−0.092	0.56
Proportion robins+sparrows	0.13	0.17	0.029	0.81	−0.045	0.77	0.21	0.064	−0.0098	0.95
Total bird abundance	−0.037	0.68	−0.023	0.86	−0.26	0.096	0.18	0.11	−0.26	0.11
Bird species richness	0.13	0.17	0.077	0.54	−0.19	0.21	0.078	0.49	−0.044	0.78

Results from a multiple regression model on effects of land-use and climate on mosquito and bird abundance and community composition.

## Discussion

The prevalence of WNV infections increased in birds (reservoir hosts) and horses (incidental hosts) across landscapes modified for orchard production and with reduced precipitation (Fig. 1). Similarly, the prevalence of WNV infections in humans was negatively associated with precipitation, although human infections were not associated with land-use. Human cases, however, may not accurately reflect counties where infections actually occurred due to traveling; the same could be true for migratory birds that travel outside of the county where they were infected.

Our results show that decreased precipitation was associated with higher prevalence of WNV infection (Fig. 2). Dry conditions can reduce the abundance of mosquito predators and competitors, leading to increased mosquito abundance and disease prevalence [31]. Dry conditions can also promote congregations of mosquitoes and birds in refuges where water is present, and dispersal from such refuges can promote disease spread [9], [32]. Conversely, high levels of precipitation can decrease adult mosquito activity and larval survival [33], [34]. Our results show that climate did not significantly affect mosquito or bird distributions (Table 1). This suggests that the effects of climate on the prevalence of WNV infections could be due to altered mosquito or bird activity, rather than a quantitative effect on vector or host abundances.

Our results show that in addition to climatic factors, land-use strongly affected the prevalence of WNV across landscapes (Fig. 1). Results seen here are generally in accordance with studies on the prevalence of WNV infection at landscape scales [8], [9]. For example, intensification of agriculture has been shown to promote the prevalence of WNV infections, measured on a per-county basis, in humans and horses [7]–[9]. Here we show that orchard habitats, but not vegetable/forage crops, were associated with a greater prevalence of WNV. Thus, our results show that not all forms of agriculture should be considered equal in terms of their suitability for promoting or limiting the spread of WNV. As agricultural management practices differ widely among regions, states, counties, and crop types, future models should explicitly test how specific types of agriculture (or other land-use factors) affect disease prevalence.

While our results suggest that land-use and climate strongly affect the prevalence of WNV infections across landscapes, infections per county may fail to reveal underlying ecological processes that operate over smaller spatial scales [35]. To address this uncertainty, we examined the prevalence of WNV infection in mosquitoes at a local scale (field locations within a county). This allowed us to determine whether greater prevalence of infection in incidental hosts (mammals) and reservoir hosts (birds) was linked with greater prevalence of WNV in mosquito vectors. Furthermore, this allowed us to increase the resolution of our estimates for infection risk at a sub-county level scale, a commonly overlooked component in epidemiological studies of arboviruses [35], [36].

Our results reveal that orchard habitats, which promoted infections in mammals and birds across landscapes, were also associated with the local prevalence of WNV infection in mosquitoes (Fig. 3). By performing spatially-explicit analyses at two spatial scales, our results linked landscape patterns of infection with local ecological factors that support pathogen transmission. For example, while natural habitats and vegetable/forage crops were associated with more WNV infections in mosquitoes in 2010, they did not affect infections in humans, horses, or birds at the landscape scale (Table 1). This suggests that these habitats did not produce focal points for WNV infection. In contrast, more abundant orchard habitats were associated with greater WNV prevalence in mosquitoes in both 2009 and 2010, and greater prevalence of WNV across landscapes (Fig. 3). This suggests that orchard habitats produced local focal points that promoted vector-host contact and subsequent pathogen transmission across landscapes, supporting the hypothesis that local processes strongly impact WNV infections across landscapes [10].

The anthropophilic nature of mosquito vectors and bird reservoirs is often assumed to be associated with the strong effects of agricultural intensification on WNV prevalence [10]. To test this hypothesis, and to determine the mechanism by which orchard habitats promoted the prevalence of WNV infection in mosquitoes, birds, and incidental hosts, we analyzed the impacts of land-use and climate on mosquito and bird communities. Our analysis focused on the two most prevalent WNV vectors in the Pacific Northwestern United States, *Cx. pipiens* and *Cx. tarsalis*, and two key bird species, the American robin and the house sparrow. Kilpatrick et al. [16] showed that even when relatively uncommon, American robins were likely associated with nearly 60% of WNV infections in mosquitoes from several locations in the eastern United States. Similarly, Hamer et al. [18] found that >95% of infectious *Cx. pipiens* mosquitoes had fed on house sparrows and/or robins; Kent et al. [17] found that in Colorado, American robins were a source of WNV infected mosquitoes early in the season and house sparrows were a key host later in the season. These seasonal dynamics are important for WNV transmission to incidental hosts, as mosquitoes often shift their feeding from bird to non-bird hosts when preferred bird hosts become less abundant [37].

Not surprisingly, orchards were associated with greater abundance of mosquitoes, American robins, and house sparrows across sites (Table 1). Orchards provide a readily available source of plant nectar during flowering, which is essential for the survival of adult mosquitoes [38]. Both American robins and house sparrows also use orchards as nesting and feeding sites [39], [40]. Thus, orchards promoted the abundance of three species that are critical components of the WNV transmission cycle. In other words, orchard habitats likely amplified vectorial capacity by promoting host-vector contact and supporting mosquito survival [41], [42]. By amplifying vectorial capacity, these habitats promoted the spread of WNV from focal points of infection across landscapes. These relationships were only identified by linking local and landscape-level processes.

Although American robins and house sparrows benefitted from orchards, we found no evidence for effects of land-use or climate on bird richness or total abundance (Table 1). The spread of infection is often reduced when host diversity is high [43], [44]. This “dilution effect” occurs because the number of host species that are unsuitable blood-meal sources, or are poor reservoir hosts for the pathogen, increases in diverse communities. This makes it less likely that vectors will feed on suitable blood-meal hosts or pathogen reservoirs [43]. However, our results suggest that the dilution effect did not occur in our study regions. In contrast, American robins and house sparrows increased in both relative and absolute abundance in orchards (Table 1). This might lead to a greater proportion of feedings on these highly-suitable reservoirs, amplifying WNV spread, which suggests these species are key for WNV transmission [16]–[18].

It is clear that predicting the spread of arboviruses requires a system-based approach that explores ecological interactions between vectors, hosts, and pathogens across landscapes [1], [10]. While the spread of many pathogens are often well characterized at landscape scales, the complex ecological factors driving these patterns at local scales are often poorly understood [35]. Here, we showed that combining spatially-explicit models with an assessment of vector, host, and pathogen distributions allows for a robust examination of the processes driving arbovirus transmission across multiple spatial scales. Linking local and landscape-level epidemiological studies in this way can form the basis for management strategies to predict and reduce the spread of arboviruses and other pathogens.

## Supporting Information

Figure S1 **Breeding bird survey sites.**
(DOCX)

Figure S2 ***R*** **-squared values from logistic regression models.**
(DOCX)

Figure S3 **Akaike’s Information Criterion (AIC) from logistic regression models.**
(DOCX)

Table S1 **The classification of each habitat type from the Cropland Datalayer maps.**
(DOCX)

Table S2 **Results of non-parametric regression analyses.**
(DOCX)

Table S3 **Results of logistic regression analyses.**
(DOCX)

## References

[1] Reisen WK (2010) Landscape epidemiology of vector-borne diseases. Annu Rev Entomol 55: 461–483. 19737082 10.1146/annurev-ento-112408-085419

[2] Weaver SC, Reisen WK (2010) Present and future arboviral threats. Antiviral Res 85: 328–345. 19857523 10.1016/j.antiviral.2009.10.008 PMC2815176

[3] Kramer LD, Styer LM, Ebel GD (2008) A global perspective on the epidemiology of West Nile virus. Annu Rev Entomol 53: 61–81. 17645411 10.1146/annurev.ento.53.103106.093258

[4] LaDeau SL, Kilpatrick AM, Marra PP (2007) West Nile virus emergence and large-scale declines of North American bird populations. Nature 447: 710–713. 17507930 10.1038/nature05829

[5] Brown HE, Childs JE, Diuk-Wasser M, Fish D (2008) Ecological factors associated with West Nile virus transmission, Northeastern United States. Emerg Infect Dis 14: 1539–1545. 18826816 10.3201/eid1410.071396 PMC2609885

[6] Ward MP, Wittich CA, Fosgate G, Srinivasan R (2009) Environmental risk factors for equine West Nile virus disease cases in Texas. Vet Res Commun 33: 461–471. 19031106 10.1007/s11259-008-9192-1

[7] Bowden SE, Magori K, Drake JM (2011) Regional differences in the association between land cover and West Nile virus disease prevalence in humans in the United States. Am J Trop Med Hyg 84: 234–238. 21292890 10.4269/ajtmh.2011.10-0134 PMC3029173

[8] Wimberly MC, Hildreth MB, Boyte SP, Lindquist E, Kightlinger L (2008) Ecological niche of the 2003 West Nile virus epidemic in the Northern Great Plains of the United States. PLoS One 3: e3744. 19057643 10.1371/journal.pone.0003744 PMC2586649

[9] Wang G, Minnis RB, Belant JL, Wax CL (2010) Dry weather induces outbreaks of human West Nile virus infections. BMC Infect Dis 10: 38. 20181272 10.1186/1471-2334-10-38 PMC2841181

[10] Kilpatrick AM (2011) Globalization, land use, and the invasion of West Nile virus. Science 334: 323–327. 22021850 10.1126/science.1201010 PMC3346291

[11] Center for Disease Control. ArboNet. Available: http://diseasemaps.usgs.gov/wnv_historical.html. Accessed 2012 October 29.

[12] Washington State Department of Health. West Nile virus surveillance maps and statistics. Available: http://www.doh.wa.gov/DataandStatisticalReports/DiseasesandChronicConditions/WestNileVirus.aspx. Accessed 2012 October 29.

[13] Darsie RF, Ward RA (2004) Identification and geographical distribution of the mosquitoes of North America, north of Mexico. Gainesville: University of Florida Press. 400 pp.

[14] Kesavaraju B, Farajollahi A, Lampman RL, Hutchinson M, Krasavin NM, et al (2012) Evaluation of a rapid analyte measurement platform for West Nile virus detection based on United States mosquito control programs. Am J Trop Med Hyg 87: 359–363. 22855771 10.4269/ajtmh.2012.11-0662 PMC3414577

[15] US Geological Survey Patuxent Wildlife Research Center. North American Breeding Bird Survey. Available: http://www.pwrc.usgs.gov/bbs/. Accessed 2012 October 29.

[16] Kilpatrick AM, Daszak P, Jones MJ, Marra PP, Kramer LD (2006) Host heterogeneity dominates West Nile virus transmission. Proc Roy Soc B 273: 2327–2333. 10.1098/rspb.2006.3575 PMC1636093 16928635

[17] Kent R, Juliusson L, Weissmann M, Evans S, Komar N (2009) Seasonal Blood-Feeding Behavior of *Culex tarsalis* (Diptera: Culicidae) in Weld County, Colorado, 2007. J Med Entomol 46: 380–390. 19351092 10.1603/033.046.0226

[18] Hamer GL, Chaves LF, Anderson TK, Kitron UD, Brawn JD, et al (2011) Fine-scale variation in vector host use and force of infection drive localized patterns of West Nile virus transmission. PLoS One 6: e23767. 21886821 10.1371/journal.pone.0023767 PMC3158794

[19] US Department of Agriculture National Agriculture Statistics Service Spatial Analysis Research Section. Cropland Data Layer. Available: http://www.nass.usda.gov/research/Cropland/SARS1a.htm. Accessed 2012 October 29.

[20] ESRI (2010) ArcGIS Desktop: Release 10. Redlands, CA.

[21] Meehan TD, Hurlbert AH, Gratton C (2010) Bird communities in future bioenergy landscapes of the Upper Midwest. Proc Nat Acad Sci USA 107: 18533–18538. 20921398 10.1073/pnas.1008475107 PMC2972996

[22] Service MW (1997) Mosquito dispersal – the long and short of it. J Med Entomol 34: 578–588. 10.1093/jmedent/34.6.579 9439109

[23] Western regional climate center. Cooperative climatological data summaries. Available: http://www.wrcc.dri.edu/climatedata/climsum/. Accessed 2012 October 29.

[24] Carrière Y, Dutilleul P, Ellers-Kirk C, Pedersen B, Haller S, et al (2004) Sources, sinks, and the zone of influence of refuges for managing insect resistance to Bt crops. Ecol Appl 14: 1615–1623.

[25] SAS Institute (2010) JMP 10.0. Cary, NC.

[26] Pelletier B, Dutilleul P, Larocque G, Fyles JW (2009) Coregionalization analysis with a drift for multi-scale assessment of spatial relationships between ecological variables 1. Estimation of drift and random components. Environ Ecol Stat 16: 439–466.

[27] Pelletier B, Dutilleul P, Larocque G, Fyles JW (2009) Coregionalization analysis with a drift for multi-scale assessment of spatial relationships between ecological variables 2. Estimation of correlations and coefficients of determination. Environ Ecol Stat 16: 467–494.

[28] Dutilleul P (2011) Spatio-Temporal Heterogeneity: Concepts and Analyses. Cambridge: Cambridge University Press. 393 pp.

[29] The Mathworks Inc (2008) MATLAB Version R2008a. Natick, MA.

[30] Carrière Y, Ellers-Kirk C, Hartfield K, Larocque G, Degan B, et al (2012) Large-scale, spatially-explicit test of the refuge strategy for delaying insecticide resistance. Proc Nat Acad Sci USA 109: 775–780. 22215605 10.1073/pnas.1117851109 PMC3271916

[31] Chase JM, Knight TM (2003) Drought-induced mosquito outbreaks in wetlands. Ecol Lett 6: 1017–1024.

[32] Shaman J, Day JF, Stieglitz M (2002) Drought-induced amplification of Saint Louis encephalitis virus, Florida. Emerg Infect Dis 8: 575–580. 12023912 10.3201/eid0806.010417 PMC2738489

[33] Paaijmans KP, Wandago MO, Githeko AK, Takken W (2007) Unexpected high losses of *Anopheles gambiae* larvae due to rainfall. PLoS ONE 2: e1146. 17987125 10.1371/journal.pone.0001146 PMC2063461

[34] Jones CJ, Lounibos LP, Marra PP, Kilpatrick AM (2012) Rainfall influences survival of *Culex pipiens* mosquitoes in a residential neighborhood in the mid-Atlantic USA. J Med Entomol 49: 467–473. 22679852 10.1603/me11191 PMC3375620

[35] Eisen RJ, Eisen L (2008) Spatial modeling of human risk of exposure to vector-borne pathogens based on epidemiological versus arthropod vector data. J Med Entomol 45: 181–192. 18402133 10.1603/0022-2585(2008)45[181:smohro]2.0.co;2

[36] Rochlin I, Ginsberg HS, Campbell SR (2009) Distribution and abundance of host-seeking *Culex* species at three proximate locations with different levels of West Nile virus activity. Am J Trop Med Hyg 80: 661–668. 19346396

[37] Kilpatrick AM, Kramer LD, Jones MJ, Marra PP, Daszak P (2006) West Nile Virus Epidemics in North America Are Driven by Shifts in Mosquito Feeding Behavior. PLoS Biology 4: e82. 16494532 10.1371/journal.pbio.0040082 PMC1382011

[38] Clements AN (2010) The Biology of Mosquitoes. Oxfordshire: CABI. 752 pp.

[39] Fluetsch KM, Sparling DW (1994) Bird nesting success and diversity in conventionally and organically managed apple orchards. Environ Toxicol Chem 13: 1651–1659.

[40] Lothrop HD, Reisen WK (2001) Landscape affects the host-seeking patterns of *Culex tarsalis* (Diptera: Culicidae) in the Coachella Valley of California. J Med Entomol 38: 325–332. 11296843 10.1603/0022-2585-38.2.325

[41] Reisen WK (1989) Estimation of vectorial capacity: relationship to disease transmission by malaria and arbovirus vectors. Bull Soc Vector Ecol 14: 39–40.

[42] Marra PP, Griffing S, Caffrey C, Kilpatrick AM, McLean R, et al (2004) West Nile virus and wildlife. Bioscience 54 393–402.

[43] Keesing F, Holt RD, Ostfeld RS (2006) Effects of species diversity on disease risk. Ecol Lett 9: 485–498. 16623733 10.1111/j.1461-0248.2006.00885.x

[44] Allan BF, Langerhans RB, Ryberg WA, Landesman WJ, Griffin NW, et al (2009) Ecological correlates of risk and prevalence of West Nile virus in the United States. Oecologia 158: 699–708. 18941794 10.1007/s00442-008-1169-9

