# Supplementary material for: West Nile Virus Prevalence across Landscapes Is Mediated by Local Effects of Agriculture on Vector and Host Communities
Source: PLoS One. 2013 Jan 30;8(1):e55006. doi: 10.1371/journal.pone.0055006 (PMC3559328; doi:10.1371/journal.pone.0055006)
Supplement: Figure S1 — Breeding bird survey sites. (DOCX) [file pone.0055006.s001.docx]

**Figure S1. Breeding bird survey sites.** Shown are Breeding Bird Survey (BBS) sites used in the analysis of bird abundance and species richness. Counties shaded gray are counties where West Nile virus (WNV) was detected in any species over this period.

**
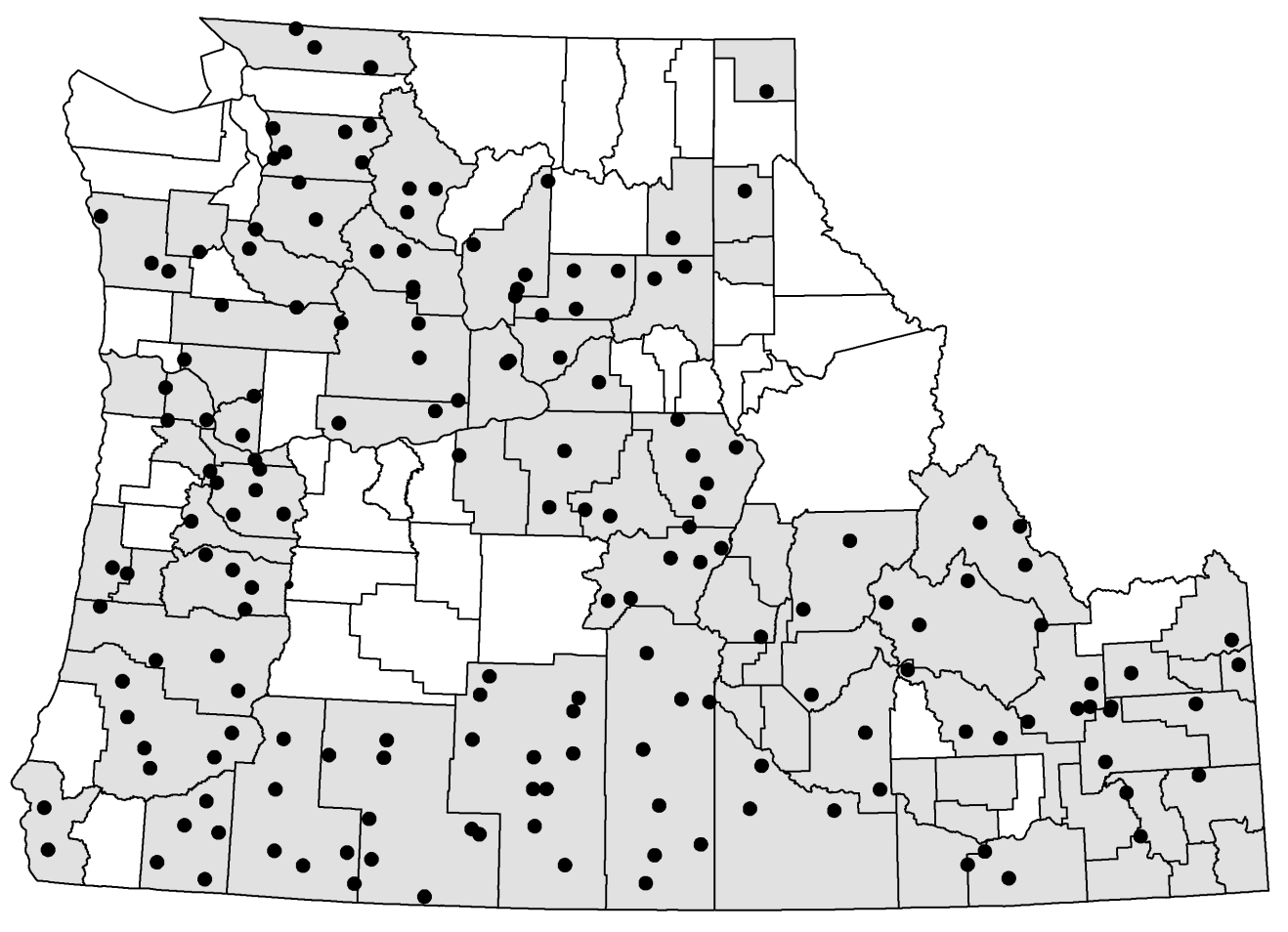
**
