# Supplementary material for: West Nile Virus Prevalence across Landscapes Is Mediated by Local Effects of Agriculture on Vector and Host Communities
Source: PLoS One. 2013 Jan 30;8(1):e55006. doi: 10.1371/journal.pone.0055006 (PMC3559328; doi:10.1371/journal.pone.0055006)
Supplement: Figure S3 — Akaike’s Information Criterion (AIC) from logistic regression models. (DOCX) [file pone.0055006.s003.docx]

**Figure S3. Akaike’s Information Criterion (AIC) from logistic regression models.** Models tested the effects of land-use and climate on prevalence of West Nile virus (WNV) infections in mosquito pools. Shown are values from models at 10 spatial scales in (A) 2009 and (B) 2010.

**
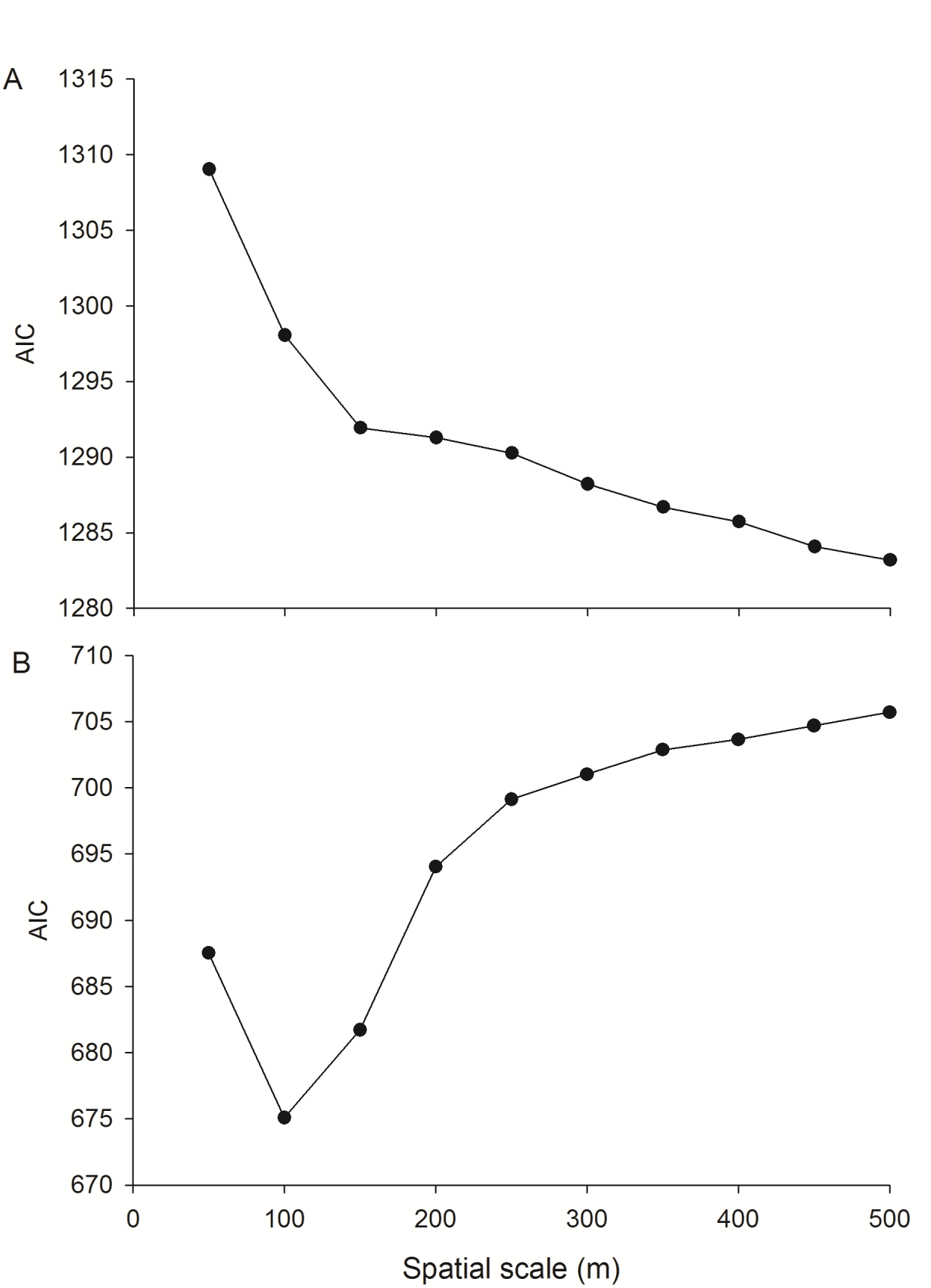
**
