# Supplementary material for: West Nile Virus Prevalence across Landscapes Is Mediated by Local Effects of Agriculture on Vector and Host Communities
Source: PLoS One. 2013 Jan 30;8(1):e55006. doi: 10.1371/journal.pone.0055006 (PMC3559328; doi:10.1371/journal.pone.0055006)
Supplement: Table S1 — The classification of each habitat type from the Cropland Datalayer maps. (DOCX) [file pone.0055006.s004.docx]

**Table S1. The classification of each habitat type from the Cropland Datalayer maps.**

| Habitat | Vegetable / forage crops | Orchard crops | Natural |
| --- | --- | --- | --- |
| Alfalfa | X |  |  |
| Apples |  | X |  |
| Apricots |  | X |  |
| Asparagus | X |  |  |
| Barley | X |  |  |
| Barren |  |  | X |
| Blueberries |  | X |  |
| Broccoli | X |  |  |
| Cabbage | X |  |  |
| Camelina | X |  |  |
| Caneberries |  | X |  |
| Canola | X |  |  |
| Carrots | X |  |  |
| Cauliflower | X |  |  |
| Cherries |  | X |  |
| Christmas trees |  | X |  |
| Clover | X |  |  |
| Corn | X |  |  |
| Cranberries |  | X |  |
| Cucumber | X |  |  |
| Deciduous forest |  |  | X |
| Developed |  |  |  |
| Dry beans | X |  |  |
| Durum wheat | X |  |  |
| Evergreen forest |  |  | X |
| Fallow | X |  |  |
| Flaxseed | X |  |  |
| Garlic | X |  |  |
| Grapes |  | X |  |
| Grassland herbaceous |  |  | X |
| Greens | X |  |  |
| Hay | X |  |  |
| Herbs | X |  |  |
| Hops | X |  |  |
| Ice / Snow |  |  | X |
| Lentils | X |  |  |
| Lettuce | X |  |  |
| Mint | X |  |  |
| Mixed forest |  |  | X |
| Mustard | X |  |  |
| Nectarines |  | X |  |
| Oats | X |  |  |
| Onion | X |  |  |
| Open water |  |  | X |
| Other crops | X |  |  |
| Other tree fruits |  | X |  |
| Other tree nuts |  | X |  |
| Pasture |  |  | X |
| Peaches |  | X |  |
| Pears |  | X |  |
| Peas | X |  |  |
| Peppers | X |  |  |
| Plums |  | X |  |
| Potatoes | X |  |  |
| Prunes |  | X |  |
| Pumpkins | X |  |  |
| Radishes | X |  |  |
| Rape Seed | X |  |  |
| Rye | X |  |  |
| Safflower | X |  |  |
| Shrubland |  |  | X |
| Sod | X |  |  |
| Sorghum | X |  |  |
| Soybeans | X |  |  |
| Speltz | X |  |  |
| Spring wheat | X |  |  |
| Squash | X |  |  |
| Strawberries |  | X |  |
| Sugarbeets | X |  |  |
| Sunflower | X |  |  |
| Sweet corn | X |  |  |
| Tomatoes | X |  |  |
| Triticale | X |  |  |
| Turnips | X |  |  |
| Vetch | X |  |  |
| Walnuts |  | X |  |
| Watermelon |  | X |  |
| Wetlands |  |  | X |
| Winter wheat | X |  |  |
| Woodland |  |  | X |
