# Supplementary material for: West Nile Virus Prevalence across Landscapes Is Mediated by Local Effects of Agriculture on Vector and Host Communities
Source: PLoS One. 2013 Jan 30;8(1):e55006. doi: 10.1371/journal.pone.0055006 (PMC3559328; doi:10.1371/journal.pone.0055006)
Supplement: Table S2 — Results of non-parametric regression analyses. (DOCX) [file pone.0055006.s005.docx]

**Table S2. Results of non-parametric regression analyses.** Analyses explored the effects of three habitat types and two climatic variables, plus all two-way interactions, on the number of West Nile virus (WNV) infections in humans, horses, and birds per county. Only the model terms retained in a stepwise regression procedure are shown. Data on human infections was standardized per capita, while data on horses and birds were standardized per unit area before analysis.

| Explanatory variable | Estimate | SE | *t* | *df* | *P* |
| --- | --- | --- | --- | --- | --- |
| *Human infections* | | | | | |
| Precipitation | -0.53 | 0.10 | -5.15 | 76 | < 0.0001 |
| *Horse infections* | | | | | |
| Orchard crops | 751.2 | 363.7 | 2.07 | 74 | 0.0001 |
| Precipitation | -0.40 | 0.098 | -4.10 | 74 | 0.042 |
| Interaction | -41.5 | 19.1 | -2.17 | 74 | 0.033 |
| *Bird infections* | | | | | |
| Orchard crops | 963.9 | 398.5 | 2.42 | 74 | 0.018 |
| Precipitation | -0.22 | 0.11 | -2.06 | 74 | 0.043 |
| Interaction | -0.55 | 21.0 | -2.64 | 74 | 0.010 |
