# Supplementary material for: West Nile Virus Prevalence across Landscapes Is Mediated by Local Effects of Agriculture on Vector and Host Communities
Source: PLoS One. 2013 Jan 30;8(1):e55006. doi: 10.1371/journal.pone.0055006 (PMC3559328; doi:10.1371/journal.pone.0055006)
Supplement: Table S3 — Results of logistic regression analyses. (DOCX) [file pone.0055006.s006.docx]

**Table S3. Results of logistic regression analyses.** Analyses examined the effects of three habitat types and two climatic variables on the prevalence of West Nile virus (WNV) infections in mosquito pools. The number of mosquitoes tested per pool was included as a covariate. Shown are results at the spatial scale explaining the most variation in incidence of WNV infections for each year (2009: 500m; 2010: 100m).

| Factor | Estimate | SE | χ^2^ | *P* |
| --- | --- | --- | --- | --- |
| 2009 | | | | |
| Mosquitoes tested | 0.074 | 0.015 | 23.2 | < 0.0001 |
| Temperature | 0.11 | 0.10 | 1.10 | 0.29 |
| Precipitation | -0.021 | 0.14 | 0.020 | 0.88 |
| Vegetable / forage crops | -7.92e-7 | 5.12e-7 | 2.40 | 0.12 |
| Orchard crops | 2.81e-6 | 1.08e-6 | 6.77 | 0.0093 |
| Natural habitats | 9.06e-6 | 4.92e-7 | 3.39 | 0.066 |
| 2010 | | | | |
| Mosquitoes tested | 0.22 | 0.038 | 34.2 | < 0.0001 |
| Temperature | -1.29 | 0.21 | 38.3 | < 0.0001 |
| Precipitation | 0.23 | 0.17 | 1.88 | 0.17 |
| Vegetable / forage crops | 0.00011 | 3.76e-5 | 8.93 | 0.0028 |
| Orchard crops | 0.00020 | 3.47e-5 | 32.8 | < 0.0001 |
| Natural habitats | 0.00014 | 2.96e-5 | 23.0 | < 0.0001 |
